# Are Health e-Mavens the New Patient Influencers?

**DOI:** 10.3389/fpsyg.2020.00779

**Published:** 2020-04-24

**Authors:** Ana M. Díaz-Martín, Anne Schmitz, María Jesús Yagüe Guillén

**Affiliations:** Departamento de Financiación e Investigación Comercial, Universidad Autónoma de Madrid, Madrid, Spain

**Keywords:** e-Mavenism, health e-Mavenism, healthcare, patient influencer, eHealth

## Abstract

Even though the healthcare industry is usually considered a rather traditional and slowly evolving sector, change is happening. Digitalization is transforming the way of obtaining medical advice and treatment and the Internet has become a key source for the seeking of healthcare information. It has allowed people to turn into more active collaborators in matters of their own health by enabling them to easily search and share information with other patients. Although research points out the growing importance of user-generated content in many sectors and its positive impact on information credibility, trust, engagement, and, ultimately, customer behavior (Malthouse et al., 2016), there is a lack of attention to this topic in healthcare. In this brief review, we address this gap by analyzing the role of health e-mavens, which are a particular type of influencers that possesses both expertise and online social influence. We lastly illustrate possible benefits of their impact on other to the different parties involved and affected by this phenomenon.

## Introduction

Traditionally, physicians have played a key role in providing health information to their patients, but, over the last few decades, there has been a considerable transformation of the forms of obtaining and retrieving medical advice. At the risk of stating the obvious, the Internet clearly is the most important disrupter and has marked a before and after for the healthcare industry. At the beginning of the 21st century, when the WWW was spreading, information provided by a personal doctor on the web and by medical universities and government sites became the most trusted sources of online health information (Dutta-Bergman, 2003). In today’s media landscape, patients are turning to the Internet also to search for peer-to-peer recommendations (Lutkenhaus et al., 2019).

Therefore, whereas in the past health information was created by doctors and used by patients, nowadays these lines become blurry as patients are increasingly in charge of their own health, collaborate with healthcare professionals rather than passively receive information from them and even create their own health recommendations for other users. This shift toward more informed, empowered and enabled patients is both desired and handy and has clear implications on the ways people interact with healthcare professionals and the healthcare system itself (Chu et al., 2017).

The Internet has become a tool to support the emergence of the educated and empowered health consumer (Powell et al., 2011) and it is frequently used by people to investigate about the meaning of symptoms, tests, diagnoses and treatments or even to have access to other peoples’ ratings, experiences, opinions or advice (O’Neill et al., 2014). Consequently, both the amount and the use of health information on the Internet are constantly growing (Dubowicz and Schulz, 2015).

In addition, new medical technologies such as electronic health records, health wearables, robotic surgery or even 3D-printing have already become part of modern healthcare, and even more innovative applications and concepts such as artificial intelligence, virtual reality or the sharing economies are no longer seen as “strangers” in the field. According to Metwally (2017), these new healthcare related technologies and the data they generate will have the same impact on the sector as Uber has had on the transport sector or Airbnb on the tourism and hospitality industry.

Therefore, in an environment where the online world meets the offline world and where interactions among patients and between them and healthcare companies are increasingly seamless and digital, there is a need to carefully study both the content that is being generated and the way people use and interpret online health information (Cuomo et al., 2020). This could be true for any industry, but it is particularly relevant for the healthcare sector, since making decisions about health issues is more complicated than choosing a pair of shoes and not all individuals are equally qualified to generate and disseminate health information. At this particular moment, for example, the spread of pseudoscience around COVID-19 through social networks has been fast and dangerous.

In summary, when it comes to healthcare, information exchange clearly is on the rise. The voice of the customer is of paramount importance. In the following section, we will review the role of user-generated content in the healthcare setting.

## User-Generated Content in Healthcare

The relevance of user-generated content is well established and recognized in different sectors. Industries such as fashion, tourism and beauty have already picked up on the importance and the impact of user generated content, exploiting and taking advantage of its impact and convenience for firms, brands and marketers (Lutkenhaus et al., 2019; Zhou et al., 2019). Staff (2018) even claims that people trust influencers more than they trust a brand and, additionally, a report by Nielsen (2015) revealed that about 83% of people completely or somewhat trust the recommendations of friends and family, and about 66% trust consumer opinions posted online.

Similar to other contexts, patients talk and exchange experiences about health problems, drug related issues and experiences and other aspects related to their health condition in different forums and social networks, where content is created, shared and used (Martínez et al., 2016). Lavorgna et al. (2017) confirm that many patients with a chronic illness rely on the Internet as their main source of information. A report published by patient-leader network Wegohealth (2018) has shown that patients are not only more likely to respond to content that comes from another patient “just like them”, but also for about 92% of the sample, online communities such as Facebook, Twitter, blogs, discussion groups and other social media sites play a significant role in their health decisions. Serving as an example in this case, a morning sickness drug recommendation on social media by influencer Kim Kardashian boosted sales by 21% (Thomas, 2019). Recent research also shows that patients find content created by other patients to be more authentic and trustworthy than content created by healthcare companies (Kanski, 2019).

But who are those individuals that are behind the healthcare content that is being created on the Internet? Eventually, anyone could post healthcare information on the WWW, but not all users are able to engage with patients and give them significant and trustworthy information. The fact that people do actively disseminate and look for health content on the web needs to be acknowledged, but it may not be forgotten that, for some people, this new role can be overwhelming and somewhat intimidating (Parker-Pope, 2008). Patients need assistance to find reliable information and, given the declining appeal of traditional health informants, such as physicians, it is important to identify active users that provide honest online health information, who are willing to share their data in order to help other patients and whose content is perceived as useful and authentic. These knowledgeable influencers are called health e mavens. Despite their relevance, there is scarce research about market mavens in social media and their influence on other patients (Cengiz et al., 2016; Agopian, 2019). A theoretical approach to this question will be offered in the next section.

## Health E-Mavens and Related Concepts

Feick and Price (1987) coined the term market mavens and defined them as consumers who have early awareness and possess high levels of knowledge and information about many products, brands, places to shop, and other facets of the market, initiate discussions with consumers and respond to requests for market information, and regularly influence people’s consumption decisions.

Therefore, market mavens are a type of influencers who pair their social influence with expertise. Influencers have a social impact, but lack expertise. Also, while content shown by influencers on social media is often sponsored and does not necessarily require personal involvement, market mavens are not driven for profit motive, they stand out by their genuine desire to help and assist others. It is important to highlight that mavens are perceived as highly reliable by other customers, which is a trait that is particularly interesting considering that people are becoming more conscious about trusting formal marketing efforts made by companies (Kiani and Laroche, 2019).

A market maven can also be seen as an opinion leader. In fact, the terms market maven and opinion leader are sometimes used interchangeably (Agopian, 2019). Nevertheless, opinion leaders are more domain specific (Dix, 2015; Casaló et al., 2018) and may have no social influence at all.

Over the years, the growing importance of online activities has led to the term e-mavens. E-mavens are the virtual version of market mavens and they have become a salient topic in the last decade, due to the shift of the channel through which information is acquired and spread by consumers and firms. They are just like market mavens except for the channel they use to communicate with other people. To date, few studies have shed some light on the demographic, psychographic and behavioral profiles of market mavens on the Internet (Belch et al., 2005; Zhang, 2010; Yang, 2013). Researchers agree that demographic variables contribute little to explain e-mavenism and demonstrate that psychographic and technological factors are significant antecedents of e-mavenism.

Combining the above mentioned concepts and applying them to healthcare, Sun et al. (2016) developed the concept of health e-mavens. They define health e-mavens as “individuals who are consistently and actively involved with health information acquisition and information transmission on the web space” (Sun et al., 2016, p. 1073). So, health e-mavens are experts that have social influence and provide general information about health goods and services on the Internet. They respond to requests from patients and actively participate in online communication and discussions. Compared to other types of influencers, they have a sense of obligation to inform patients and they enjoy helping others. Above all, they are not perceived as an elite or exclusive group of opinion leaders.

According to Sun et al. (2016), the health e-maven construct consists of two factors: one known as information acquisition (it includes the tracking of health information on various devices and consulting, meaning checking online rankings or reviews of doctors, hospitals or drugs) and another one described as information transmission (it is based on people’s active behavior of posting, for example, online rankings or reviews of doctors, hospitals and drugs and sharing health information on social media platforms). In this seminal research work, the authors designed a Likert-type scale to measure health e-mavenism and empirically validated its behavioral dimensions. The health e-maven construct opens interesting research opportunities to be further developed, since it promises theoretical and practical insights for health scholars and managers in the age of new and social media technologies.

## Conclusion and Discussion

Healthcare systems all around the world are undergoing huge changes. Patients are not only more demanding, but they are also becoming familiar with new ways of dealing with healthcare information in online and offline environments. Nowadays, markets widely acknowledge the importance of opinion leaders, influencers and market mavens as intermediaries and boosters of information exchange on the Internet.

The aim of this short review is to relate the concept of health e-mavens (Sun et al., 2016) to the framework of user-generated content in the healthcare sector and their potential to act as patient influencers. Since they combine both the potential to influence and the necessary expertise to do so, they should be considered by all stakeholders as important individuals with whom to collaborate and also an interesting research topic.

Knowing that user-generated content in healthcare is practically still in its infancy, it is important to investigate the specific profiles of health eMavens: what characteristics distinguish them? Are those antecedents similar all over the world? Are health and technology literacy relevant factors in explaining the variance in health e-mavenism? Does altruism stimulate users to engage in an e-maven like behavior?

In times where “fake news” and hoaxes spread like wildfire, it is crucial to assure the quality and the integrity of the information that is shared over the internet. Future research should monitor the content generated by health e-mavens combining data mining, machine learning and qualitative research techniques to analyze their materials. It would also be interesting to address the effects of such content on those who consume it and on healthcare professionals: does it affect their intention to spread eWOM? How do patients and physicians use the content created by e-mavens in their search of well-being? Both the patients and the health service providers’ perspectives need to be better understood.

Health e-mavens represent, as well, a critical group for health promotion practitioners to recruit and mobilize in various web-based health intervention programmers or online health communication campaigns. Recognizing the decisive influence of health e-mavens and collaborating with them is crucial for healthcare organizations trying to develop a successful social media strategy. How do firms identify them? And how should they appeal to them?

In our opinion, health managers, social media administrators, health e-mavens, data scientists, marketing researchers and influencer agencies could benefit from working together on these relevant questions.

## Author Contributions

Authors are listed in alphabetical order and have all made a substantial contribution to this manuscript.

## Conflict of Interest

The authors declare that the research was conducted in the absence of any commercial or financial relationships that could be construed as a potential conflict of interest.
